# ASO Author Reflections: Can Targeted Nutrition Level the Playing Field? Reflections on Preoperative Support for Malnourished Colorectal Cancer Patients in ERAS

**DOI:** 10.1245/s10434-025-18832-2

**Published:** 2025-12-11

**Authors:** Nuria Valdés

**Affiliations:** 1 Endocrinology and Nutrition Department, Diabetes and Endocrine Research Group, Biobizkaia Health Research Institute, Osakidetza, Hospital Universitario Cruces, Barakaldo, Spain; 2 Faculty of Medicine and Dentistry, University of the Basque Country (EHU), Endo-ERN, CIBERDEM, CIBERER, Health Institute Carlos III, Madrid, Spain

## Past

Preoperative malnutrition is a well-established risk factor for adverse outcomes after colorectal cancer surgery.^1,2^ However, evidence regarding the true impact of targeted nutritional interventions within multimodal pathways such as enhanced recovery after surgery (ERAS) has been limited and sometimes contradictory.^3,4^ The key clinical question in our study was whether a “screen-and-treat” approach—systematic screening followed by targeted nutritional support—could truly neutralize the excess surgical risk in malnourished patients or whether malnutrition would remain a negative prognostic factor despite perioperative optimization.^5^

## Present

Our prospective cohort study shows that, within a structured ERAS program, systematic identification and personalized nutritional support for malnourished patients is associated with postoperative outcomes (complications and length of stay) that are comparable to those of well-nourished peers.^5^ These findings reinforce the importance of integrating nutritional screening and targeted interventions as a core component of ERAS protocols and suggest that, when adequately addressed, malnutrition no longer predicts poorer surgical outcomes in this context.

## Future

Further multicenter studies with larger sample sizes are needed to validate these findings, including cost-effectiveness analyses and long-term follow-up. Future research should also define the optimal duration of preoperative and postoperative nutritional support and develop objective tools to monitor adherence and to capture the functional impact of nutritional interventions on overall patient recovery.
